# Prevalence, common helminthes, and factors associated with helminthes among pregnant women attending antenatal clinic at a tertiary hospital in Uganda

**DOI:** 10.1371/journal.pntd.0012926

**Published:** 2025-03-25

**Authors:** Fowsia Ali Said, Emmanuel Okurut, Naima Bashir Mohamed, Simon Byonanuwe, Richard Mulumba, Isaac Kusolo

**Affiliations:** Department of Obstetrics and Gynecology, Kampala International University-Western Campus, Bushenyi, Uganda; Central University of Tamil Nadu, INDIA

## Abstract

**Background:**

Helminthes in pregnant women is among the neglected tropical diseases. The Uganda ministry of health adopted the WHO recommendation of routine biannual deworming for girls and women of reproductive age and twice in pregnancy during the second and third trimesters. Despite the measures put in place, the prevalence of Helminthes among pregnant women in Uganda is still high which has implications for both the mother and to the developing fetus.

**Methods:**

This was a hospital-based cross-sectional study carried out from January to April, 2024. Using Consecutive sampling method, 334 pregnant women were enrolled. Data was collected using pre-tested questionnaires, and a single stool specimen was collected from each woman and freshly voided stool specimens was directly examined microscopically. The data was analyzed using STATA Version 14.2. A bivariate and multivariate analysis were used to show the association between the dependent and independent variables, considering P < 0.05 as the level of significance and the 95% confidence interval.

**Results:**

The overall prevalence of Helminthes was 27.54%. Among all pregnant women who tested positive, common helminth was Hook worm (83.7%), followed by Ascaris lumbricoides (31.5%), and Trichuris triciuria (21.7%). Age, rural residence, having no toilet facility, no hand washing after toilet use, walking bare footed, no hand washing before meals were significantly associated with Helminthes with (aOR = 0.2; 95% CI = [0.085-0.588]; P = 0.002), (aOR = 9.0; 95% CI = [1.684-48.325]; P =0.010), (aOR = 3.6; 95% CI = [1.788-7.101]; P = 0.001), (aOR = 4.7; 95% CI = [1.359-16.419]; P = 0.015), (aOR = 1.9; 95% CI = [1.014-3.674]; P = 0.045), (aOR = 13.1; 95% CI = [5.146-33..578]; P = 0.001), respectively.

**Conclusion:**

In this study, the overall prevalence of Helminthes was low in pregnancy compared to the global prevalence. The common helminthes among pregnant women was Hook worm infestation. The infection was independently associated with respect to Age, rural residence, having no toilet facility, no hand washing after toilet use, walking bare footed, no hand washing before meals.

## Author summary

Helminth infections are parasitic worm infections that are common in low- and middle-income countries, especially in areas with poor sanitation and hygiene practices. Pregnant women are particularly vulnerable to these infections, which can lead to significant health issues for both the mother and the unborn child, including anemia and complications during pregnancy. Despite the World Health Organization’s recommendation for routine deworming during pregnancy, many women in Uganda and similar settings continue to be affected by helminth infections due to gaps in public health interventions.

In this cross-sectional study, we investigated the prevalence, common helminths, and associated factors among 334 pregnant women attending antenatal care at a tertiary hospital in Uganda. We found that 27.5% of participants were infected, with hookworm being the most common parasite. Key risk factors included rural residence, lack of access to toilet facilities, walking barefoot, and poor hand hygiene before meals. These findings emphasize the urgent need for community-wide deworming campaigns, improved access to sanitation, and strengthened hygiene education as part of antenatal care services.

Our study sheds light on the current burden of helminth infections in pregnant women and highlights practical recommendations for public health interventions to improve maternal and neonatal health outcomes.

## Background

Helminthes refer to parasitic diseases caused by nematode worms [1]. There are two main phyla: nematodes (roundworms) and platyhelminths (flatworms). Nematodes include important intestinal worms and filarial worms, while platyhelminths include flukes and tapeworms [1]. Helminthes are transmitted through eggs in human feces, contaminating soil in areas with poor sanitation [2]. Pregnant women in endemic areas are particularly vulnerable, facing increased morbidity and mortality [3]. The prevalence of Helminthes is higher in pregnant women compared to non-pregnant women [3,4].

Over 100 countries are endemic to helminths, with the highest numbers in Sub-Saharan Africa, the Americas, and Asia [5]. The prevalence of hookworm among pregnant women ranges from 1% to 78% [6]. In low- and middle-income countries (LMICs), intestinal parasitic infections are neglected tropical diseases, with pregnant women experiencing a prevalence of 24% to 70% and polyparasitism rates around 10% [7].

In Sub-Saharan Africa, approximately one-third of pregnant women are infected with helminths [8], with prevalence estimates ranging from 11% to 31% [9,10]. Prevalence varies widely across countries: 11.1% in Benin [11], 25.7% in Ghana [12] and 49% in Gabon [13].

In East Africa, the pooled prevalence of helminths among pregnant women is 38.54% [14]. In rural Western Kenya, the prevalence of intestinal geohelminthiases is 13.8%, with pregnant women particularly vulnerable [15]. In Uganda, a cohort study within the Entebbe Mother and Baby Study found that 68% of women had helminths [16].

## Materials and Methods

### Ethics statement

Ethical approval for the study was obtained from the Research and Ethics Committee of Bishop Stuart University **(BSU-REC-2023-119)**. Privacy and confidentiality were ensured by individually assessing participants, anonymizing questionnaires with number codes, and securely storing data. Written informed consent was obtained after thoroughly explaining the study details to participants, with signatures or fingerprints collected. All our study participants were at least 18 years old, and no pregnant women younger than 18 years were included in our study.

### Study design and setting

This cross-sectional study was conducted at the obstetrics department of Fort Portal Regional Referral Hospital, located in Fort Portal. The hospital has a bed capacity of 384 [17] and serves the entire Tooro region, which includes eight Ugandan districts (Bundibugyo, Kabarole, Kyenjojo, Kasese, Kamwenge, Kyegegwa, Bunyangabu, and Ntoroko) and part of eastern Democratic Republic of Congo (DRC). Fort Portal Regional Referral Hospital provides both general and specialized medical services and serves as a teaching center for KIU Medical School and several other institutions.

#### Participants.

We included all pregnant women attending the antenatal clinic at Fort Portal Regional Referral Hospital during the study period, provided they gave consent to participate. We excluded women who failed to provide stool samples and those who had received a dewormer in the preceding four weeks.

#### Variables and data sources.

Eligible participants were appropriately consented, and information on socio-demographics, behavioural, and environmental characteristics was collected by the PI and trained research assistants using pretested interviewer-administered structured questionnaires and laboratory forms. The questionnaire had four sections: Section A captured socio-demographic data, while the other three sections covered specific study objectives. The reliability of the data collection tools was confirmed with a Cronbach’s coefficient alpha test score of 88%, and validity was ensured through precise and consistent instrumentation.

For stool sample collection by certified laboratory personnel, a single stool specimen was collected from each woman. Freshly voided stool specimens were directly examined microscopically and preserved with 10% formalin for further analysis. Preserved specimens were processed using the formalin-ether concentration technique and examined microscopically for ova and larvae of helminths. A direct saline and iodine wet mount of each sample was used to detect helminths microscopically [18]. Findings were reported on a laboratory report form, and participants received their results from the laboratory along with appropriate care.

### Sample size calculation

Sample size calculation formula by Leslie Kish [19] was be used to determine the Sample size for this study, using the estimated prevalence of 68% based a study in Uganda by Ndibazza [16] and the calculated sample size was 334 participants.

### Data analysis

Data analysis for this study was performed using STATA software version 14.2, structured to address three primary objectives. The prevalence of Helminthes was calculated as a fraction of women found with Helminthes divide by the total number of women recruited in the study. The findings were presented as a percentage with its corresponding 95% confidence interval. To analyze the common Helminthes, the different Helminthes observed among was described in terms of frequencies and percentages with their corresponding 95% confidence intervals. Data was presented using tables and figures like graphs accompanied by narratives. Binary logistic regression analysis was employed to analyze the relationship between factors and Helminthes, Logistic regression analysis was applied to eliminate any confounding factors associated with Helminthes. Variables with P<0.20 level at bivariate analysis were considered in the multivariate analysis so as to identify independent factors with p ≤ 0.05.

## Results

### Characteristics of study participants

A total of 334 pregnant women participated in this study, and their stool samples were analyzed for helminths. The participants’ ages ranged from 18 to 45 years, with the majority aged between 20 and 29 years (35.9%). Most participants were from urban areas (80.8%), married (94.0%), and had attended primary school (64.4%). The majority were Christians (68.3%) and farmers (50.0%). Regarding obstetric characteristics, most were between 13 and <28 weeks of amenorrhea (33.8%), had attended ANC less than 2 times (36.53%), and were multipara (43.71%) (Table 1).

**Table 1:** Socio-demographic and obstetric characteristics of the study participants (N = 334).

Variable	Category	Frequency(n)	Percentage (%)
**Socio-demographic characteristics**			
Age	18-19	41	12.3
	20-29	120	35.9
	30-39	114	34.1
	≥ 40	59	17.6
Residence	Urban	270	80.8
	Rural	64	19.2
Marital status	Single	20	6.0
	Married	314	94.0
Level of education	Uneducated	**73**	21.9
	Primary	215	64.4
	Secondary and higher	46	13.7
Religion	Christian	228	68.3
	Muslim	106	31.7
Occupation	Farmer	167	50.0
	Business	67	20.1
	Employed	53	15.8
	Unemployed	47	14.1
**Obstetrics characteristics**			
Gestation age	< 13 WOA	90	27.0
	13 – < 28 WOA	113	33.8
	≥ 28WOA	131	39.2
ANC attendance visits	< 2	122	36.6
	2–4	115	34.4
	> 4	97	29.0
Parity	Nullipara	100	29.9
	Primipara	88	26.4
	Multipara	146	43.7
History of anthelminthic use	Yes	166	49.7
	No	168	50.3

Additionally, 50.3% had no history of anthelminthic use. In terms of environmental and behavioural characteristics, the majority sourced their water from wells (44.0%) and had toilet facilities (63.5%). However, many did not wash their hands after toilet use (58.7%) or use soap (62.8%). A significant number ate unwashed fruits and vegetables (58.7%) and drank unboiled water (57.9%). Most did not walk barefoot (70.4%), washed hands before meals (59.88%), and did not have a habit of eating soil (50.6%) (Table 2).

**Table 2:** Environmental characteristics of the study participants (N = 334).

Variable	Category	Frequency(n)	Percentage (%)
Water source	Spring	109	32.6
	Well	147	44.0
	Tap	78	23.4
Having toilet facility	Yes	212	63.5
	No	122	36.5
Hand washing after toilet use	Yes	138	41.3
	No	196	58.7
Use of soap to wash hands	Yes	125	37.4
	No	209	62.6
Eating unwashed fruits and vegetables	Yes	196	58.7
	No	138	41.3
Type of drinking water	Unboiled	193	57.8
	Boiled	53	15.9
	Treated Tap water	88	26.1
Walking bare footed	Yes	99	29.6
	No	235	70.4
Hand washing before meals	Yes	200	59.9
	No	134	40.1
Habit of eating soil	Yes	165	49.4
	No	169	50.6

### Prevalence of helminthiasis among pregnant women

**Fig 1:**
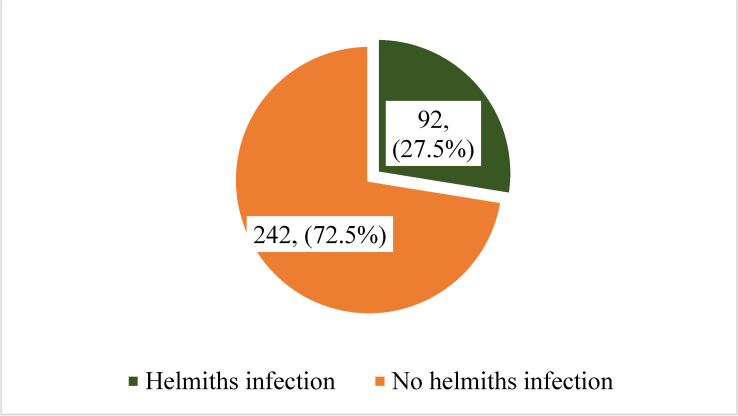
Prevalence of helminthes among pregnant women.

Out of 334 pregnant women tested, 92 of them had Helminthiasis giving an overall Prevalence of 27.5% (Fig 1).

### Common helminths among pregnant women

**Fig 2:**
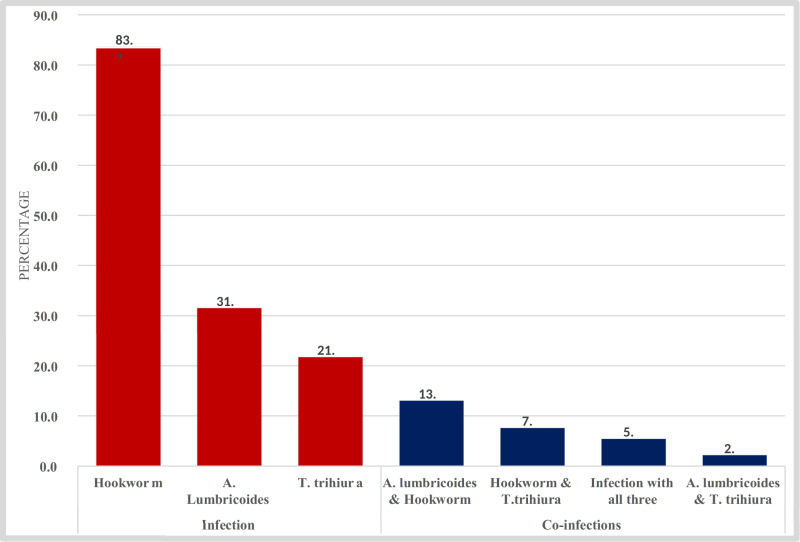
Most common helminthes of helminthes among pregnant women.

Among all pregnant women who tested positive, common helminth was Hook worm 53(58%), followed by Ascaris lumbricoides 8 (9%), Trichuris triciuria 6 (7%) then both Hook worm and Ascaris lumbricoides 12(12%), Hook worm and Trichuris triciuria 7 (6%), Ascaris lumbricoides and Trichuris triciuria 2 (2%) and all Helminthes were found in 5 (5%) of pregnant women.in this study. (Fig 2)

### Factors associated with helminthiasis among pregnant women

The bivariate analysis results indicated that three socio-demographic factors were significantly associated with helminth infections (P ≤ 0.2). These factors included age groups 20-29 (cOR = 0.3; 95% CI = [0.131-0.582]; P = 0.001), 30-39 (cOR = 0.3; 95% CI = [0.147-0.654]; P = 0.002), and ≥ 40 (cOR = 0.4; 95% CI = [0.153-0.820]; P = 0.015); being a farmer (cOR = 2.6; 95% CI = [1.187-5.701]; P = 0.017); and residence (cOR = 2.7; 95% CI = [1.280-5.746]; P = 0.009). Obstetrical factors significantly associated with helminth infections among pregnant women included a history of antihelminthic use (cOR = 2.5; 95% CI = [1.504-4.098]; P = 0.001). Environmental factors significantly associated with helminth infections included not having a toilet facility (cOR = 2.8; 95% CI = [1.715-4.608]; P = 0.001), no hand washing after toilet use (cOR = 2.2; 95% CI = [1.301-3.676]; P = 0.003), no use of soap to wash hands (cOR = 2.5; 95% CI = [1.471-4.413]; P = 0.001), eating unwashed fruits and vegetables (cOR = 1.5; 95% CI = [0.888-2.406]; P = 0.136), walking barefoot (cOR = 1.7; 95% CI = [1.027-2.840]; P = 0.039), no hand washing before meals (cOR = 2.0; 95% CI = [1.217-3.217]; P = 0.006), and the habit of eating soil (cOR = 1.7; 95% CI = [1.032-2.727]; P = 0.037). (Table 3)

**Table 3:** Bivariate analysis of socio-demographic, obstetric and environmental factors associated with Helminthes among pregnant women (N = 334).

Variable	Category	Stool test results		cOR (95% CI)	p
		No helminths (%), n=242	Helminths (%), n=92		
Age	18-19	20 (48.78)	21 (51.22)	3.6 (1.713-7.636)	0.001*
	20-29	93 (77.50)	27 (22.50)	1.00	
	30-39	86(75.44)	28(24.56)	1.1(0.612-2.052)	0.710*
	≥40	43(72.88)	16(27.12)	0.4(0.626-2.623)	0.497*
Residence	Urban	55(85.94)	9(14.06)	1.00	
	Rural	187(69.26)	83(30.74)	2.7(1.280-5.746)	0.009*
Marital status	Unmarried	12(60.00)	8(40.00)	1.8(0.721-4.62)	0.204
	Married	230(73.25)	84(26.75)	1.00	
	Tertiary	7(87.50)	1(12.50)	1.00	
Level of education	Secondary	32(84.21)	7(15.79)	1.31(0.136-12.70)	0.814
	Primary	154(71.63)	61(28.37)	2.8(0.334-23.01)	0.345
	Uneducated	49(67.12)	24(32.88)	3.4(0.39929.476)	0.262
Religion	Muslim	80(75.47)	26(24.53)	1.00	
	Christian	162(71.05)	66(28.95)	1.3(0.740-2.123)	0.401
Occupation	Formal employee	44(83.02)	9(16.98)	1.00	
	Informal employee	165(70.51)	69(29.49)	2.0(0.946-4.416)	0.069*
	Unemployed	33(70.21)	14(29.79)	2.1(0.801-5.369)	0.133*
Gestation age	<13WOA	50(55.56)	40(44.44)	2.7(1.463-4.897)	0.001*
	13-<28WOA	87(76.99)	26(23.01)	1.00	
	≥28WOA	105(80.15)	26(19.85)	0.8(0.449-1.530)	0.548
ANC attendance visits	>4	82(84.54)	28(24.35)	1.00	
	<2	73(59.84)	15(15.46)	3.7(1.899-7.091)	<0.001*
	2-4	87(75.65)	28(24.35)	1.8(0.877-3.529)	**0.112**
Parity	Nullipara	76(76.00)	24(24.00)	1.00	
	Primipara	64(72.73)	24(27.27)	1.2(0.616-2.289)	0.608
	Multipara	102(69.86)	44(30.14)	1.2(0.765-2.438)	0.291
History of antihelminth use	Yes	135(81.33)	31(18.67)	1.00	
	No	107(63.69)	61(36.31)	2.5(1.504-4.098)	<0.001*
Water source	Spring	81(74.31)	28(25.69)	1.3(0.667-2.691)	0.412
	Well	99(67.35)	48(32.65)	1.9(0.982-3.594)	**0.057**
	Tap	62(79.49)	16(20.51)	1.00	
Having toilet facility	Yes	170(80.19)	42(19.81)	1.00	
	No	72(59.02)	50(40.98)	2.8(1.715-4.608)	<0.001*
Hand washing after toilet use	Yes	112(81.16)	26(18.84)	1.00	
	No	130(66.33)	66(33.67)	2.2(1.301-3.676)	0.003*
Use of soap to wash hands	Yes	104(83.20)	21(16.80)	1.00	
	No	138(66.03)	71(33.97)	2.5(1.471-4.413)	0.001*
Eating unwashed F&V	No	106(76.81)	32(23.19)	1.00	
	Yes	136(69.39)	60(30.61)	1.5(0.888-2.406)	**0.136**
Type of drinking water	Boiled	42(79.25)	11(20.75)	1.00	
	Un-boiled	129(66.84)	64(33.16)	1.9(0.914-3.924)	0.086
	Treated Tap water	71(80.68)	17(19.32)	0.9(0.391-2.137)	0.836
Walking barefooted	No	178(75.74)	57(24.26)	1.00	
	Yes	64(64.65)	35(35.35)	1.7(1.027-2.840)	**0.039**
Hand washing before meals	Yes	136(69.39)	60(30.61)	1.00	
	No	106(76.81)	32(23.19)	2.0(1.217-3.217)	0.006*
Habit of eating soil	No	131(77.51)	38(22.49)	1.00	
	Yes	111(67.27)	54(32.73)	1.7(1.032-2.727)	**0.037**

F & V: fruits and vegetables, cOR: Crude Odds Ratio, CI: Confidence Interval

At multivariate analysis, several factors were significantly associated with helminth infections in pregnancy. These included age (aOR = 0.2; 95% CI = [0.085-0.588]; P = 0.002), rural residence (aOR = 9.0; 95% CI = [1.684-48.325]; P = 0.010), having no toilet facility (aOR = 3.6; 95% CI = [1.788-7.101]; P = 0.001), no hand washing after toilet use (aOR = 4.7; 95% CI = [1.359-16.419]; P = 0.015), walking barefoot (aOR = 1.9; 95% CI = [1.014-3.674]; P = 0.045), and no hand washing before meals (aOR = 13.1; 95% CI = [5.146-33.578]; P = 0.001). (Table 4)

**Table 4:** Multivariate analysis of factors associated with Helminthes among pregnant women attending antenatal clinic at fort portal regional referral hospital (N = 334).

Variable	Category	cOR(95%CI)	P	aOR(95%CI)	P
**Having a toilet facility**	Yes	1.00		1.00	
	No	2.8(1.715-4.608)	<0.001*	5.6(2.600-12.194)	<0.001**
**Age**	18-19	3.6 (1.713-7.636)	0.001*	4.3 (1.575-11.690)	0.004**
	20-29	1.00		1.00	
	30-39	1.1 (0.612-2.052)	0.710*	1.9 (0.830-4.132)	0.133
**Residence**	Urban	1.00		1.00	
	Rural	2.7 (1.280-5.746)	0.009*	10.4 (2.462-44.130)	0.001**
**Occupation**	Formal employee	1.00		2.0 (0.946-4.416)	0.069
	Informal employee	2.0 (0.946-4.416)	0.069*	0.3 (0.054-1.312)	0.104
	Unemployed	2.1 (0.801-5.369)	0.133*	2.5 (0.622-10.452)	0.194
**Gestation age**	<13WOA	2.7 (1.463-4.897)	0.001*	0.9(0.320-2.562)	0.852
	13- 28WOA	1.00		1.00	
	≥28WOA	0.8 (0.449-1.530)	0.548	0.9(0.344-2.105)	0.727
**ANC attendance visits**	>4	1.00		1.00	
	<2	3.7 (1.899-7.091)	<0.001*	1.9(0.532-7.019)	0.316
	2-4	1.8 (0.877-3.529)	0.112*	1.0(0.375-2.454)	0.930
**History of antihelminth use**	Yes	1.00		1.00	
	No	2.5 (1.504-4.098)	<0.001*	4.6(1.505-14.059)	0.007**
**Water source**	Spring	1.3(0.667-2.691)	0.412	13.9(3.498-55.329)	<0.001**
	Well	1.9(0.982-3.594)	**0.057**	3.12(0.884-11.012)	0.077
	Tap	1.00		1.00	
**Hand washing after toilet use**	Yes	1.00		1.00	
	No	2.2(1.301-3.676)	0.003*	4.1(1.21-14.14)	0.024**
**Use of soap to wash hands**	Yes	1.00		1.00	
	No	2.5(1.471-4.413)	0.001*	6.9(2.06-22.98)	0.002**
**Eating unwashed F&V**	No	1.00		1.00	
	Yes	1.5(0.888-2.406)	**0.136**	0.4(0.13-1.09)	0.072
**Walking barefooted**	No	1.00		1.00	
	Yes	1.7(1.027-2.840)	**0.039**	1.8(0.92-3.41)	0.088
**Hand washing before meals**	Yes	1.00		1.00	
	No	2.0(1.217-3.217)	0.006*	19.7(7.37-52.81)	<0.001**
**Habit of eating soil**	No	1.00		1.00	
	Yes	1.7(1.032-2.727)	**0.037**	0.9(0.46-1.83)	0.813

cOR: Crude Odds Ratio, CI: Confidence Interval, P**: ≤ 0.05, aOR = adjusted odds ratio.

## Discussion

### Prevalence of helminthes among pregnant women

The overall prevalence of helminths among pregnant women in this study was 27.0%. This finding aligns with studies by Tesfaye et al. [20], which reported a prevalence of 29.5%, and a study in Ethiopia with a prevalence of 24.7% [21]. However, the prevalence in this study was lower than that reported in a meta-analysis in the USA, which ranged from 1% to 78% [6], and other studies such as Hailu et al. [22] at 37.3%, a study in Portugal at 55.9% [23], Nigeria at 34.2% [24]), Maytsebri primary hospital at 51.5% [1], and a meta-analysis in East Africa at 38.54% [25]. The differences could be due to larger sample sizes in these studies. Conversely, the prevalence in this study was higher than that found in the USA at various stages of ANC [26], Burkina Faso at 1.3% [27], Ghana at 14.3% [28], Kenya at 12.4% [29], and a cross-sectional study in Lira district, Uganda at 11% [30]. These differences may be attributed to smaller sample sizes in some studies and varying standards of living and education levels across differing economic zones.

### Common helminthes among pregnant women

Among all pregnant women who tested positive, the most common helminth was hookworm, followed by Ascaris lumbricoides and Trichuris trichiura. This finding is consistent with studies by Hailu et al. [22], Mengist et al. [21], Yesuf et al. [31], and Anunobi et al. [24], which also identified hookworm as the leading cause of intestinal parasitosis, followed by Ascaris lumbricoides and Trichuris trichiura. However, a study in Portugal found Ascaris lumbricoides to be the most predominant species (90.9%), followed by Trichuris trichiura (13.8%) and polyparasitism (11.9%) [23]. Similar findings were reported by Feleke & Jember [4], Alula et al. [8], and Abaka et al. [28] in Ghana, where Ascaris lumbricoides was the predominant species. These differences could be attributed to variations in environmental factors and lifestyles between these countries that favor different helminths.

### Factors associated with helminthiasis among pregnant women

In this study, age was significantly associated with helminth infections in pregnant women, aligning with findings from other studies, such as a systematic review and meta-analysis by Aemiro et al [32] and research in Ethiopia by Feleke & Jember [4]. Younger age groups are often involved in activities and behaviors that increase their risk of helminthic infections. Rural residence was 9.0 times more likely to be associated with helminthiasis, similar to findings from studies by Aemiro et al. [32], Hailu et al. [22], Feleke & Jember [4], Tesfaye et al. [20], and Mosisa et al. [25] in East Africa. This is because rural women often engage in activities like digging, which exposes them to soil helminths, and hygiene standards are generally lower compared to urban areas.

Having no toilet facility was 3.6 times more likely to be associated with helminthiasis, consistent with findings from studies by Aemiro et al. [32], Hailu et al. [22], Mengist et al. [21], Feleke & Jember [4], and Mosisa et al. [25]. Open defecation leads to contamination of food and water sources with helminths. Not washing hands after toilet use was 4.7 times more likely to be associated with helminth infections, as shown in studies by Feleke & Jember [4], Dagnaw et al. [33], and Yesuf et al. [31]. Contaminated hands can spread gastrointestinal helminths if not washed properly.

Walking barefoot was 1.9 times more likely to be associated with helminth infections, similar to findings from studies by Aemiro et al. [32], Feleke & Jember [4], Dagnaw et al. [33], Tesfaye et al. [20], and Yesuf et al. [31]. Soil worms can easily come into contact with the human body when walking barefoot. Not washing hands before meals was 13.1 times more significantly associated with helminth infections in pregnancy, as shown in studies by Feleke & Jember [4]. This is mainly due to contamination.

### Study strength and limitation

This is the first study in Ugandan history to record the incidence of common helminths and the risk factors for the disease in expectant mothers. The primary strength of this study was that it was conducted by licensed healthcare professionals who strictly adhered to data collection protocols and used conventional tools and techniques (a microscope). However, the results of this institutionally based cross-sectional study might not be generalizable to the entire population.

### Conclusions and recommendation

The study found a relatively low prevalence of helminths among pregnant women compared to global rates. Hookworm was the most common infection, with significant associations to factors such as age, rural residence, lack of toilet facilities, walking barefoot, and not washing hands before meals. The study recommends that the Ugandan Ministry of Health implement preconception care to provide antihelminth medication to all women of childbearing age. Health facilities, including Fort Portal Regional Referral Hospital, should prioritize treating hookworm and educate pregnant women on the risk factors and preventive measures for helminth infections.

## Supporting information

S1 File Questionnaire used for data collection during the study, covering socio-demographic, environmental, and behavioral factors. (DOCX)

S2 File Raw data on helminth prevalence, co-infections, and associated factors used in statistical analyses. (XLS)
