# Supplementary material for: Prevalence, common helminthes, and factors associated with helminthes among pregnant women attending antenatal clinic at a tertiary hospital in Uganda
Source: PLoS Negl Trop Dis. 2025 Mar 25;19(3):e0012926. doi: 10.1371/journal.pntd.0012926 (PMC11936221; doi:10.1371/journal.pntd.0012926)
Supplement: S1 File — (DOCX) [file pntd.0012926.s001.docx]

## APPENDIX IV: Questionnaire

**Topic:** Prevalence, common Helminthes and associated factors of Helminthes among pregnant women attending antenatal clinic at Fort portal regional referral hospital.

|  | Date of interview | | | | | | | | |  | | | |  | | Phone number | | | | | | | | | |
| --- | --- | --- | --- | --- | --- | --- | --- | --- | --- | --- | --- | --- | --- | --- | --- | --- | --- | --- | --- | --- | --- | --- | --- | --- | --- |
|  |  |  | | |  | | | | |  | | |  | |  |  |  | | | | | | | |  |
| **Soc iodemographic factors** | | | | | | | | | |  | | | | | |  | | | | | | | | | |
| 1. | Age  How old are you? ……… | | | | | | | | |  | | | | 2. | | Education level | | | | | | | | | |
|  |  |  |  |  |  |  |  |  |  |  |  |  |  |  |  | Uneducated Primary secondary tertiary/university | | | | |  | |  | | |
|  |  |  |  |  |  |  |  |  |  |  |  |  |  |  |  |  |  |  |  |  |  | |  |  |  |
|  |  |  |  |  |  |  |  |  |  |  |  |  |  |  |  |  |  |  |  |  |  | |  |  |  |
|  |  |  |  |  |  |  |  |  |  |  |  |  |  |  |  |  |  |  |  |  |  | |  |  |  |
| 3. | Religion | | | | | | | | | …………. | | | | 4. | | Occupation | | | | | | | | | |
|  | Pegan  Christian Muslim  Other specify | | |  | | | ………… | | |  |  |  |  |  |  | Unemployed  Employed | | | | |  | | |  | |
|  |  |  |  |  | | |  |  |  |  |  |  |  |  |  |  |  |  |  |  |  | | |  |  |
|  |  |  |  |  | | |  |  |  |  |  |  |  |  |  |  |  |  |  |  |  | | |  |  |
|  |  |  |  |  | | |  |  |  |  |  |  |  |  |  |  |  |  |  |  |  |  |  |  |  |
| 5. | Marital status | | | | | | | | | …………… | | | | 6. | | Residence | | | | | | | | | |
|  | Single  Married  Other specify | | |  | | | ……… | | |  |  |  |  |  |  | Rural  Urban | | | |  | |  | | | |
|  |  |  |  |  | | |  |  |  |  |  |  |  |  |  |  |  |  |  |  | |  |  |  |  |
|  |  |  |  |  | | |  |  |  |  |  |  |  |  |  |  |  |  |  |  | |  |  |  |  |
|  |  |  |  |  | | |  |  |  |  |  |  |  |  |  |  |  |  |  |  | |  |  |  |  |
| **Ob stetrical factors** | | | | | | | | | |  | | | | | |  | | | | | | | | | |
| 1. | Gestational age……… | | | | | | | | |  | | | | 2. | | Parity…………………………….. | | | | | | | | | |
| 3. | ANC attenda nce visits | | | | | | | | |  | | | | 4. | | Previous history of antihelminthic use | | | | | | | | | |
|  | < 4 visits  ≥ 4 visits | | | |  | | |  | |  |  |  |  |  |  | Yes  No | | |  | |  | | | | |
|  |  |  |  |  |  | | |  |  |  |  |  |  |  |  |  |  |  |  | | | | | | |
| **En vironmental factors** | | | | | | | | | |  | | | | | |  | | | | | | | | | |
| 1. | Water source | | | | | | | | |  | | | | 2. | | Have toilet facility | | | | | | | | | |
|  | Tap  Well  Spring | | |  | | |  | | |  |  |  |  |  |  | Yes  No | | |  | |  | | | | |
|  |  |  |  |  | | |  |  |  |  |  |  |  |  |  |  |  |  |  | |  |  |  |  |  |
|  |  |  |  |  | | |  |  |  |  |  |  |  |  |  |  |  |  |  | |  |  |  |  |  |
|  | Other specify …………………………  …………………………… | | | | | | | | | | | | |  | |  | | | | | | | | | |
| 3. | Have toilet facility | | | | | | | | | | | | | 4. | | Wash hands after toilet | | | | | | | | | |
|  | Yes  No | |  | | |  | | | | | | | |  |  | Yes  No | |  | | |  | | | | |
|  |  |  |  | | |  |  |  |  |  |  |  |  |  |  |  |  |  | | |  |  |  |  |  |
| 5. | Use soa p to wash hands | | | | | | | | | | | | | 6. | | Eat unw ashed fruits and vegetables | | | | | | | | | |
|  | Yes  No | |  | | |  | | | | | | | |  |  | Yes  No | |  | | |  | | | | |
|  |  |  |  | | |  |  |  |  |  |  |  |  |  |  |  |  |  | | |  |  |  |  |  |
|  |  |  |  | | |  |  |  |  |  |  |  |  |  |  |  |  |  | | |  |  |  |  |  |
| 7. | Type of drinking water | | | | | | | | | | | | | 9. | | Walk ba re fo oted | | | | | | | | | |
|  | Well/stream/river (unboiled) | | | | | | | | | |  |  | |  |  | Yes  No | |  | | |  | | | | |
|  |  |  |  |  |  |  |  |  |  |  |  |  |  |  |  |  |  |  | | |  |  |  |  |  |
|  | Tap water  Boiled water  Treated water | | | | | | | |  |  |  |  |  |  |  |  |  |  | | |  |  |  |  |  |
|  |  |  |  |  |  |  |  |  |  |  |  |  |  |  |  |  |  |  | | |  |  |  |  |  |
|  |  |  |  |  |  |  |  |  |  |  |  |  |  |  |  |  |  |  |  |  |  |  |  |  |  |
|  |  |  |  |  |  |  |  |  |  |  |  |  |  |  |  |  |  |  |  |  |  |  |  |  |  |
| 10. | Hand washing before meal | | | | | | | | | | | | | 11. | | Habit of eating soil | | | | | | | | | |
|  | Yes  No | |  | | |  | | | | | | | |  |  | Yes  No | |  | | |  | | | | |
|  |  |  |  | | |  |  |  |  |  |  |  |  |  |  |  |  |  | | |  |  |  |  |  |
|  |  |  |  | | |  |  |  |  |  |  |  |  |  |  |  |  |  | | |  |  |  |  |  |

**Checklist (for the principal Investigator Only)**

**Stool test results**

Helminths infection

No helminths infection

If helminths infection, specify the intestinal parasite species

……………………………………………………………………………………… ……………………………………………………………………………………………………… …………………………………………………………………………………………………….

……………………………………………………………………………………………..
